# Life is in motion (through a chick’s eye)

**DOI:** 10.1007/s10071-022-01703-8

**Published:** 2022-10-12

**Authors:** Bastien S. Lemaire, Giorgio Vallortigara

**Affiliations:** Center for Mind and Brain Sciences, University of Trento, Trento, Italy

**Keywords:** Motion perception; animacy; predispositions, Domestic chicks, Human newborns

## Abstract

Cognitive scientists, social psychologists, computer scientists, neuroscientists, ethologists and many others have all wondered how brains detect and interpret the motion of living organisms. It appears that specific cues, incorporated into our brains by natural selection, serve to signal the presence of living organisms. A simple geometric figure such as a triangle put in motion with specific kinematic rules can look alive, and it can even seem to have intentions and goals. In this article, we survey decades of parallel investigations on the motion cues that drive animacy perception—the sensation that something is alive—in non-human animals, especially in precocial species, such as the domestic chick, to identify inborn biological predispositions. At the same time, we highlight the relevance of these studies for an understanding of human typical and atypical cognitive development.

## Introduction

Motion plays a vital role in the detection and communication of animals, because it drives actions. Remarkably, animals can detect and even interpret actions with simplified motion displays. Point-light animations representing an animal’s articulations motion were introduced by Johansson (1973), showing that they can convey the essential information to detect simple actions, such as walking. 27 years later, in one of the first volumes published by Animal Cognition, Regolin et al. (2000) reported that domestic chicks were sensitive to such biological motion. A few years earlier, Blake (1993) and Omori and Watanabe (1996) reported that cats and pigeons could discriminate between a biological motion pattern and controlled versions of it (either containing similar point-light displays with an altered temporal sequence or by presenting the biological motion patterns of distinct species). Still, these studies used associative learning, where animals require substantial training and experience with stimuli. In contrast, Regolin et al. (2000) used a different form of learning based on exposure only, i.e., filial imprinting. This process occurs in the first days of many precocial species, and in just a few hours of exposure to a stimulus, inexperienced animals form a strong attachment and differentiate it from others (Vallortigara and Versace 2018). Contrary to associative learning, imprinting is driven by some sort of 'instinct' to find the most suitable stimulus. A stimulus that must represent its mother, provides shelter and food and teaches them how to thrive and survive in their environment (Bateson 1990; Rosa-Salva et al. 2015; Vallortigara 2021).

Many naive biases have been discovered when imprinting was scrutinised in the laboratory. Some shapes, colours, structures and motions appeared to catch the chicks’ attention more quickly than others (Cate 1989; Hoffman 1978; Johnson and Horn 1988; Kovach 1971; Lemaire 2020; Lemaire et al. 2021; Schulman et al. 1970). Biases have been discovered in naïve, newly hatched domestic chicks that appear to canalise their attention toward animacy cues and lead them to approach stimuli that are more likely to be social partners (Di Giorgio et al. 2017a, b; Rosa-Salva et al. 2015; Vallortigara 2012, 2021). The use of a precocial species has allowed scientists to document these evolutionary-given preferences (predispositions) in neonate chicks having received no specific prior experience; something that would be nearly impossible to investigate in altricial species. Indeed, in the laboratory, chicks can hatch in perfectly controlled environments and be tested immediately after that. They hatch ready to explore their environment and are equipped with inborn predispositions to find the most suitable stimulus to imprint on: something that is alive (Rosa-Salva et al. 2015; Vallortigara 2021). We could then wonder: what are the motion properties of something that is alive (sometimes dubbed animacy perception), and what can domestic chicks tell us about it? The study performed by Regolin et al. (2000) acted as a starting point for many experiments performed in domestic chicks and from which today's journey began—a dive across decades of parallel investigations on the motion cues driving animacy perception in animals while highlighting the relevance of studying precocial species, such as domestic chicks. In this review article, we focus on the motion cues that evoke animacy perception—the sensation that something is alive. The literature discussed here will be intentionally mainly directed toward studies using domestic chicks, as it has proven to be an ideal animal model to approach this topic from the onset of life. Implications of the findings for parallel studies conducted in human adults and newborns will also be stressed. Note that we use terms such as instinct or inborn/naive biases/predispositions or evolutionary-given preferences (predispositions) to denote mechanisms or traits selected through natural selection as they provide a selective advantage. Some of those inborn biases can be species-specific or not, present at birth or appear after non-specific experiences or during a sensitive period (Rosa-Salva et al. 2015, 2021; Vallortigara 2021). They reveal a sort of general abstract scaffolding or building blocks on which further social knowledge can be built.

## Motion drives animacy

The motion of a living animal is important to notice. Let us take a simple example, where the branches and leaves of a tree are moving. It might be windy. This does not provide us with beneficial information for our survival. It might also be that a bird is moving on that tree branch and causing its motion. Noticing and categorising this motion signature as being caused by a living creature is of greater importance. It could be a congener, a predator, or a delicious meal, something that could directly impact our survival.

### Animacy revealed by its cause

For some scientists, to identify animacy from motion cues, we should base our decision upon inferences related to the causes of motion (Gelman et al. 1995; Tremoulet and Feldman 2000) and the following assumption: when the cause of motion is internal, self-generated, it is more likely to be alive; if the cause is external, it is more likely not to be alive (Vallortigara 2012). In our previous example, the branches’ and leaves’ motion can be caused by the wind (external). They swing and eventually fall to the ground due to gravity and are more likely to be inanimate. In fact, human adults perceive a stimulus moving down as less animate than a stimulus moving up (Szego and Rutherford 2008). Moving up, changing directions, speed and starting to move from rest—as the bird moving on the branch of our example could do—requires an internal source of energy that most animals possess and is, as a result, more likely to be animate. In line with this idea, scientists have studied simple motion cues that imply an internal energy source and whether such signals are used to detect and categorise objects as animate.

**Fig. 1:**
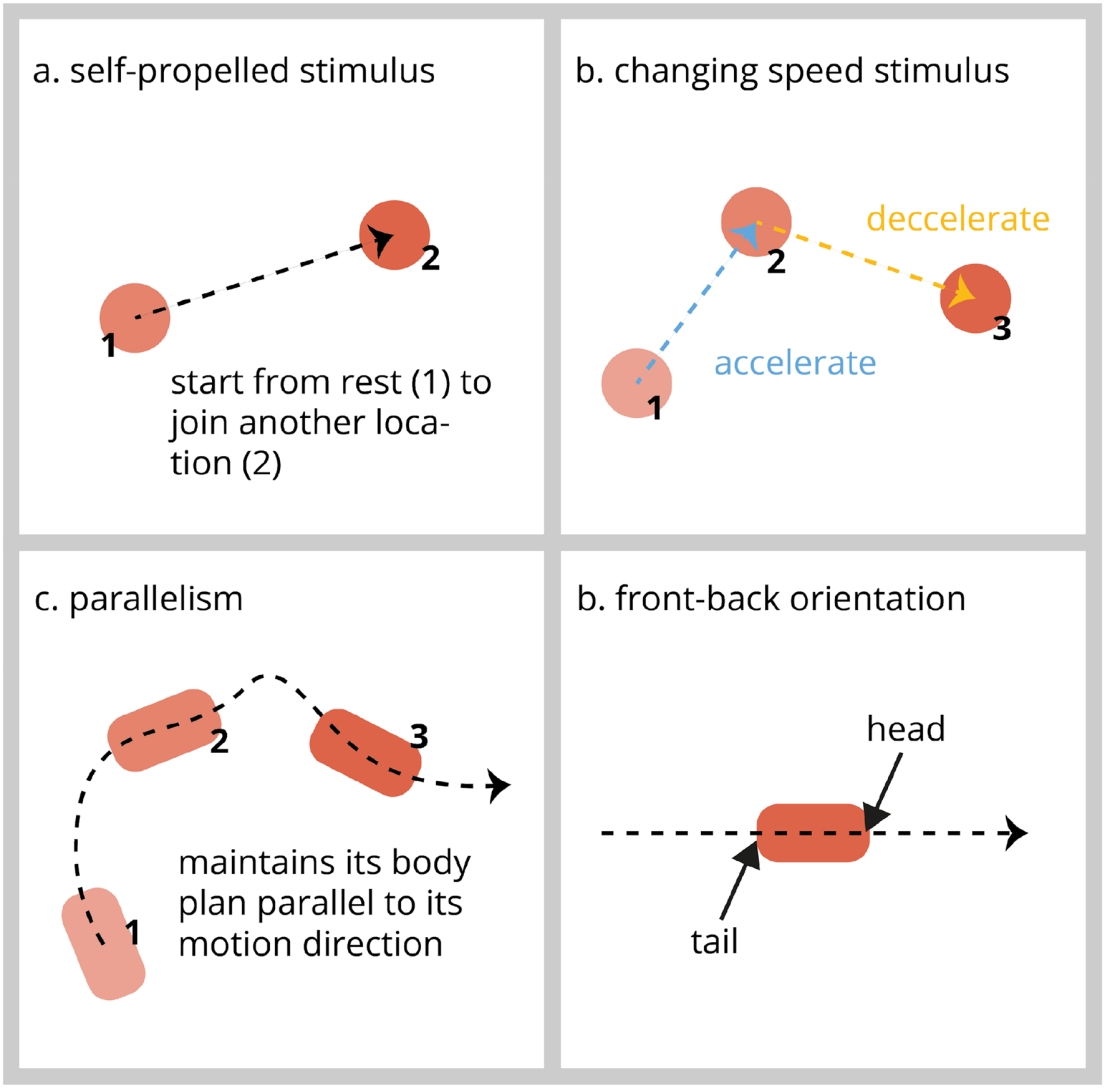
Motion cues of a single stimulus driving animacy perception. **a** Stimulus starts to move on its own and **b** changes speed suggesting it possesses an internal energy source. **c** Stimulus maintains its main body axis parallel to its direction. **d** Stimulus moving in a direction provides information on the location of its front and back orientation

Tremoulet and Feldman (2000) asked human participants to give animacy rankings to simple dots changing speed and direction while keeping their main body axis aligned with their motion direction (parallelism): three motion cues that imply an internal source of energy (see Fig. 1). The authors reported that animacy ratings were strongest with significant changes in speed and direction and objects maintaining their alignment with the motion direction. Thus, it seems that simple objects with such motion features convey animacy perception in adults. Interestingly, this is also true in human newborns that can differentiate between self- and non-self-propelled objects (Di Giorgio et al. 2017a, b) as well as objects that change speed in different ways (Di Giorgio et al. 2021a, b). Such evidence suggests the existence of inborn predispositions to visual cues of motion that trigger animacy perception.

Research on domestic chicks aligns with such conclusions. Mascalzoni et al. (2010) wondered whether imprinted chicks would prefer to associate with self-propelled objects (implying an internal energy source, Fig. 1a) or objects put in motion through physical contact (external energy source). Remarkably, chicks preferred to associate with the self-propelled object. Similarly, in other studies, chicks chose to associate with a changing speed object (Fig. 1b) rather than an object moving at a constant speed (Rosa-Salva et al. 2016), a preference that has now been replicated twice (Lorenzi et al. 2021; Versace et al. 2019). Moreover, Rosa-Salva et al., (2018) reported that naive chicks spontaneously preferred rotational motion, which could indicate self-propulsion and, therefore, would align with previous findings. These results obtained in human newborns and newly hatched chicks, suggest the existence of inborn preferences for visual cues of motion that trigger animacy perception. Furthermore, those inborn evolutionary-given predispositions appear to be shared across taxa and have probably evolved from common vertebrate ancestors. Therefore, one could wonder what other motion signatures represent living creatures in our visual systems.

### Body axes and motion direction

Most animals (prehistoric and modern) have a bilateral body plan (Knoll and Carroll 1999), and their locomotions are constrained by it. In most cases, animals keep their main body axis aligned with their motion direction (see Fig. 1c). Imagine going down a street and seeing everybody walking sideways. That would be odd. We could also assume that a moving object keeping its main body axis aligned with its direction would be more likely to be perceived as an animate entity than an inanimate one. As suggested earlier, this is apparent in adult humans who attribute higher animacy scores to simple stimuli that keep their main body axis aligned with their motion direction (Tremoulet and Feldman 2000). Interestingly, this sort of motion is also preferred by visually naive chicks that spontaneously associate with them (Rosa-Salva et al. 2018). What is also interesting about the maintenance of body alignment with the motion direction is that it provides a cue to determine the front-back orientation of an animal—the position of the head and the tail. Most animals, in fact, travel head first (as depicted in Fig. 1d), making it a piece of valuable information. For example, male red-sided garter snakes use the females’ motion direction to locate themselves adequately during courting (Shine et al. 2000). Another example can be found in toads that only snap at a bar when this is moving horizontally but not vertically (Ewert 1987 and 2004). We can observe similar behaviours in newly hatched chicks that spontaneously peck at an elongated shape moving in relation to their main body axis (Clara et al. 2009). Moreover, 6-month-old infants rapidly encode the axial direction of novel agents to predict their future behaviour (Hernik et al. 2014). The body structure constrains motion, and therefore, motion induced by it becomes representative of animate beings.

### Biological motion

**Fig. 2:**
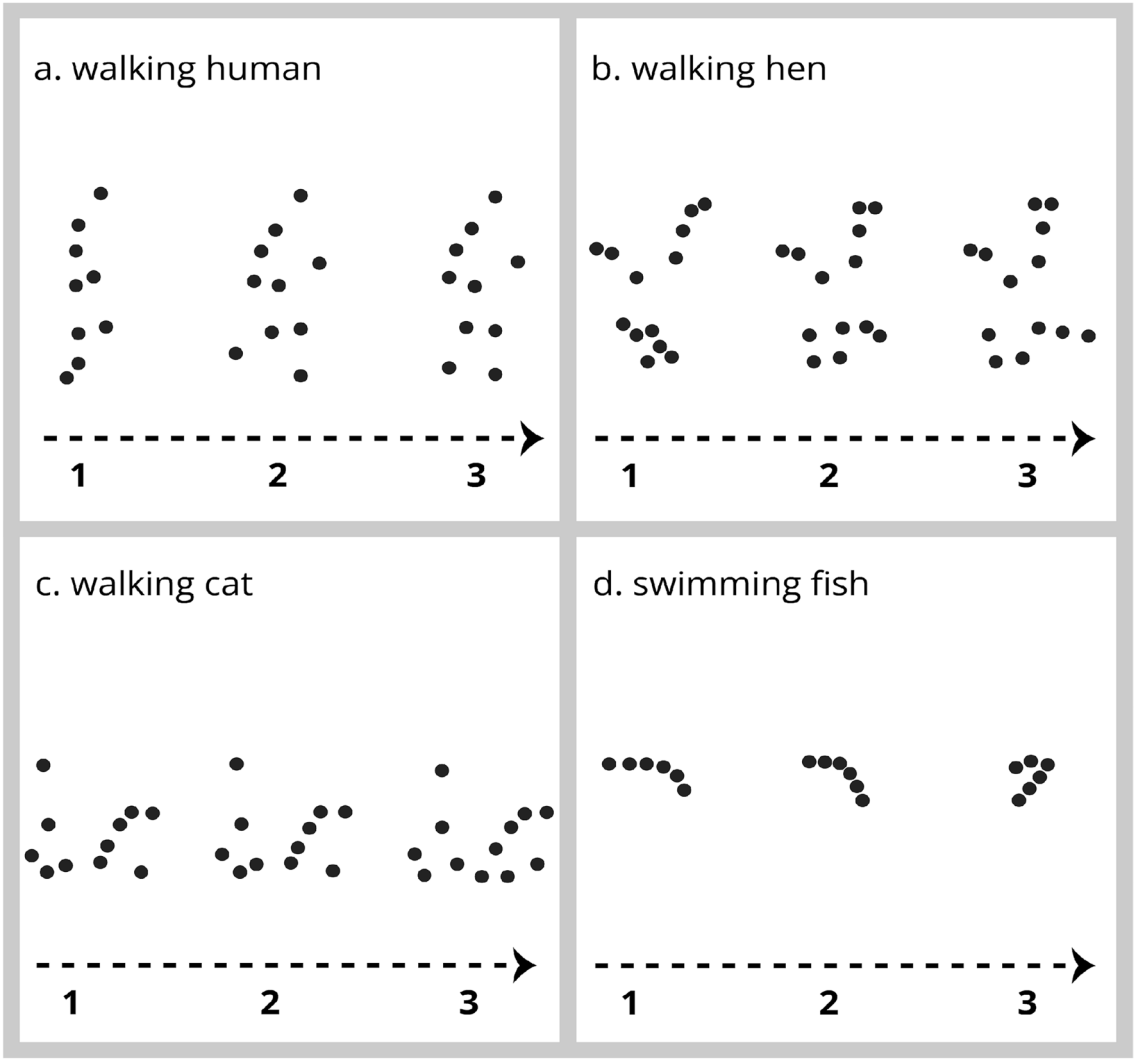
Examples of biological motion patterns representing the motion and actions of different species (numbers represent different frames)

Now, let us go back to the starting point of this review article: biological motion. Biological motion has a particular signature dictated by the animal’s body structure (the biological motion of a walking cat will be different from the one of a walking hen, see Fig. 2, for examples of biological motion patterns) and the action it mimics (flying will be very different from walking). Biological motion patterns carry the essential information of several human locomotory actions such as walking, jumping, dancing and boxing (Dittrich 1993) as well as socially relevant information such as gender (Mather and Murdoch 1994), affect (Pollick et al. 2001), personality traits (Heberlein et al. 2004), and identity (Jokisch et al. 2006; Troje et al. 2005). Those actions and socially relevant information appear evident to an experienced eye, but what would they mean to a naïve one?

Vallortigara et al. (2005), addressed this issue in naïve individuals finding that newly hatched chicks spontaneously associated with a biological motion of a walking hen in comparison with controlled motion patterns (a rigid motion pattern, where a single frame of the point light walking hen animation was taken and moved vertically along the screen or a random motion pattern when point lights moved in arbitrary directions). Even more interestingly, when given a choice between the biological motion of a walking hen and a walking cat, chicks did not show any preference; both patterns indeed appeared to be equally attractive to them. This suggests that chicks' preference for biological motion was not species-specific and, therefore, not specific to an animal's body structure, but probably more related to the motion rules of living animals that are similar across the animal kingdom. When in action, the point-light displays are synchronised and move in relation to one another. Some points are anchored to others and keep a constant distance; some points are not and get closer or further away as the action continues (e.g., the left ankle point will always hold the same distance from the left knee point during walking but will get closer to the hip points when the paw rises). Those properties make biological motion patterns look alive. A few years later, Simion et al. (2008) confirmed the findings in chicks and reported that 2-day-old babies preferentially look at a biological motion display compared to a non-biological one. Since then, many other species, such as spiders (De Agrò et al. 2021) and different species of fish (Larsch and Baier 2018; Nakayasu and Watanabe 2014; Schluessel et al. 2015; Shibai et al. 2018), have been tested and appear to discriminate biological from non-biological motion patterns. Shibai et al. (2018) also suggested that the preference for biological motion pattern was led by the posture elements of the point light and/or the motion trajectory in a 3D environment.

Until now, we have described causal motion properties of single agents that trigger animacy perception. However, in a social context, when animals interact, new motion cues trigger animacy perception and might even reveal intentions.

## Motion drives social interactions

Let us return to our bird hiding in its tree. Now, imagine two birds hiding and potentially interacting with each other in the same tree. Observing how they move might tell us more about their intentions. They might engage in a nuptial display, play, or chase each other as if one were a predator and the other its prey. Therefore, one might wonder what kind of motion cues identify them as alive beings and, from there, help us understand their interactions.

In the pioneering works of Heider and Simmel (1944) and Michotte (1963), simple shapes put in motion together led to powerful animacy percepts and the attribution of intentions. Of course, these motion patterns were manually created and thus not carefully controlled, which stimulated other researchers to design more controlled experiments to investigate such innate preferences.

Among all the motion sequences, a specific display involving multiple agents produced a strong animacy perception: chasing. Chasing has been the topic of many investigations in human infants (Frankenhuis et al. 2013; Rochat et al. 1997, 2004) and adults (Barrett et al. 2005; Frankenhuis et al. 2013; Gao et al. 2009; Gao and Scholl 2011; Van Buren et al. 2016) and helped to unravel motion cues that reveal the presence of living animals and potentially their intentions.

### Spatiotemporal contingencies

**Fig. 3:**
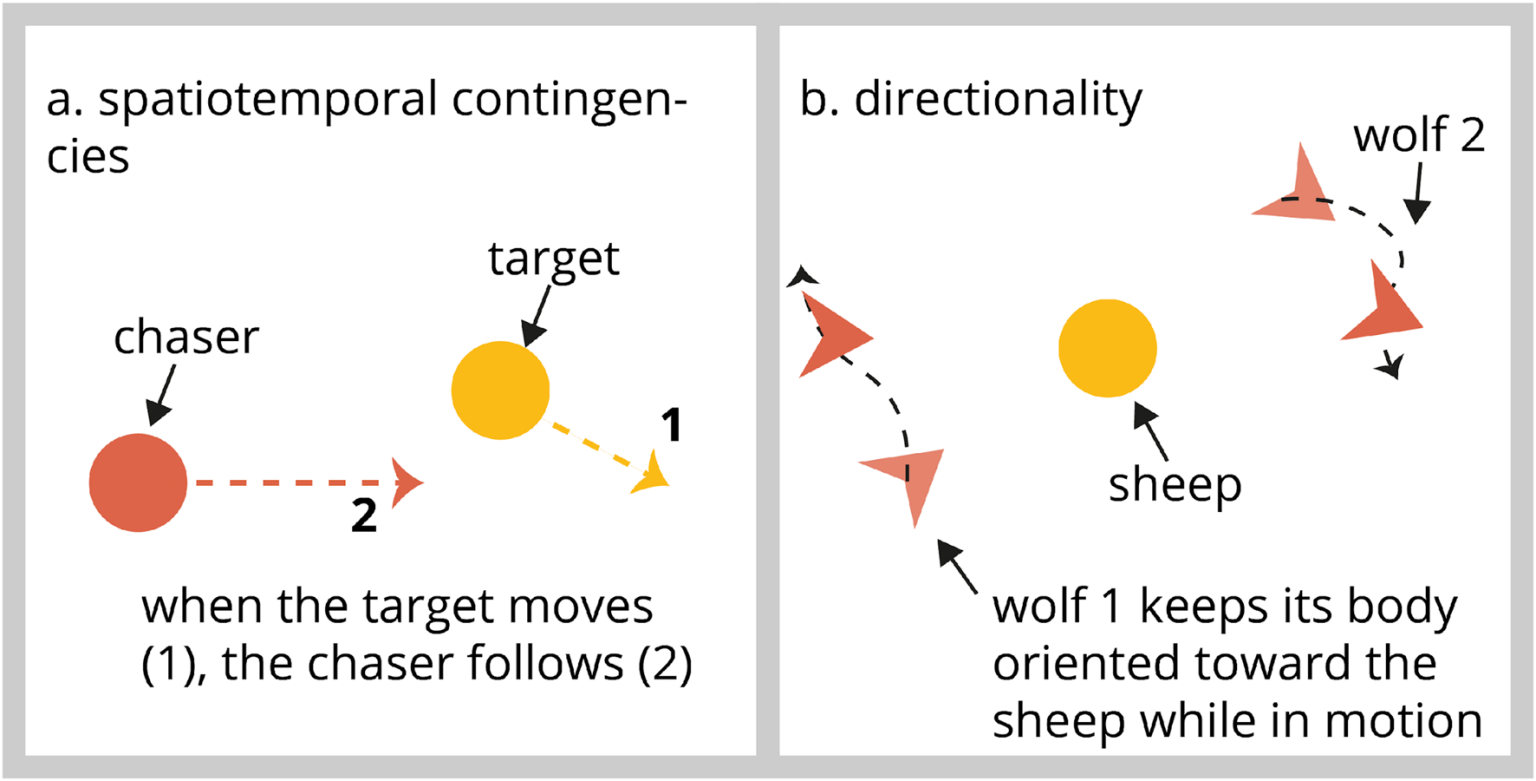
Motion cues of interacting agents driving animacy perception. **a** Spatiotemporal contingencies of two agents engaged in a chase. **b** Agent’ directionality (wolf 1) when chasing its target (sheep)

During a chase, one agent follows and approaches another agent. Therefore, both agents are linked in time and space as the chaser gets closer to its target with time (Fig. 3a). Bassili (1976) prepared five computer-generated films to test the effect of spatiotemporal contingencies on animacy perception in human adults and found that the temporal component was crucial for the perception of an interaction between agents, while the spatial component tended to determine its nature. Later, Dittrich and Lea’s study (1994) contradicted Bassili’s generalisation as they demonstrated (using moving letters) that both spatial and temporal parameters were essential for detecting and interpreting a chase. In a slightly different study, focusing on the importance of the context on animacy perception, Tremoulet and Feldman (Tremoulet and Feldman 2006) demonstrated that the location of a static dot more or less close to the path of another moving dot significantly influenced the degree to which animacy was perceived. Indeed, in this experiment, participants were more likely to think that both dots were linked when they were closer. Interestingly, reducing the distance between two objects attracts the attention of infants’ and adults’ eyes (Dittrich and Lea 1994; Galazka and Nyström 2016; Meyerhoff et al. 2014). In recent work, we tested whether visually naïve chicks would respond to agents whose motions were reciprocally contingent in space and time (Lemaire et al. 2022). While chicks did not prefer spatially contingent agents, they paid attention to the temporal contingencies and preferred agents that moved in a temporally unpredictable manner. This demonstrates that, prior to any visual experience, chicks can use an essential component of social interaction events.

### Directionality

Another essential feature of chasing is the directionality of the chase—the chaser always faces its target and maintains its orientation toward it. Gao et al. (2010) demonstrated this by manipulating the orientation of darts (termed wolves) chasing a dot (termed sheep) and asking participants to detect the presence of a chase in a motion display (Fig. 3b). When the wolf was facing the sheep, the chase was quickly spotted. However, when it was not, the participant’s performance in finding it drastically decreased. This was termed “the wolfpack effect” and demonstrates that directionality is a relevant cue to perceived animacy. More interestingly, this feature is relevant for perceived animacy irrespective of a chase’s temporal and spatial components. Indeed, darts oriented in the direction of a dot appeared to interact with it, although in reality the darts moved in random directions (Gao et al. 2010).

### Leaving room for variability

Chasing events are mostly composed of spatiotemporal contingencies between two agents and their related directional information. Those cues drive animacy perception alone or mixed in a motion display. Although the perfect chase appears to be a “heat-seeking” pursuit—the chaser pursues its target while facing it and taking the shortest path and the minimum time possible—the perception of chasing accepts a certain degree of freedom. The path of the chaser does not necessarily need to be the shortest and can slightly deviate (Gao et al. 2009). However, the more it varies, the less obvious chasing becomes. Interestingly, the amount of interruption during a chase does not impair its detection (Gao and Scholl 2011). In contrast, when the periods of interruptions are replaced with random or local motion, the detection performance of the chase decreases rapidly (Gao and Scholl 2011).

Allowing for variability when detecting a chase, or more generally animacy, appears essential when placed in a natural context. Predators are not continually adopting a “heat-seeking” plan but might use other strategies. For example, the tentacle snake rounds around its prey, predicting its escape response (Eaton et al. 1977), so that the prey ends up in its mouth (Catania 2011). Detecting chasing is crucial for most animals (from hunting prey, avoiding predators, mating, etc.) but only a few studies have investigated how non-human animals perceive it. A first attempt was made by Goto et al. (2002) by training pigeons to discriminate chasing from non-chasing patterns and vice-versa. Although the pigeons never reached the learning criterion, they showed consistent discrimination between the patterns. Atsumi and Nagasaka (2015) made a similar attempt and successfully trained squirrel monkeys, using an associative learning procedure, to discriminate chasing from randomly moving shapes on a screen. After that, Abdai and colleagues (Abdai et al. 2017a, b; Abdai et al. 2021; Abdai et al. 2017a, b; Abdai and Miklósi 2022) showed that dogs could spontaneously differentiate between chasing-like and independent motion patterns without any training. They first reported that dogs paid more attention to chasing-like events. However, in subsequent studies using different stimuli and procedures, Abdai and colleagues reported that dogs paid more attention to the independent motion patterns suggesting that the experimental settings might slightly influence the directions of the preference. It is interesting to notice that a similar shift in attention is observed in human infants in the first few months of life (Rochat et al. 1997).

Although we have recently demonstrated that visually naïve chicks extract and use temporal contingencies (Lemaire et al. 2022), whether the detection of chasing per se is innate (biologically predisposed) or acquired through experience remains to be determined. This opens the way for exciting further research taking advantage of the domestic chick’s eyes, filled as they are with the knowledge of evolution. In the meantime, not only one but multiple motion components, such as the spatiotemporal contingencies or directionality of agents, appear helpful for detecting and interpreting specific actions of living entities; a faculty that is available early in life and independent from enculturation (Barrett et al. 2005; Rochat et al. 2004).

## Neural correlates of animacy perception

Since the innovative motion displays of Heider and Simmel (1944) and Johansson (1973), researchers have wondered which brain regions are responsible for the perception and attribution of animacy.

Several studies performed in humans with the use of PET and fMRI scanning have focused on point-light displays mimicking specific actions, such as dancing-like motion and even more localised and goal-directed actions, such as a hand reaching for a glass, picking it up and bringing it to the mouth. Overall, these studies have reported the implication of parts of the right posterior superior temporal sulcus and fusiform gyrus (Bonda et al. 1996; Grossman et al. 2000; Lichtensteiger et al. 2008; Peelen et al. 2006; Vaina et al. 2001) as well as in the adjacent middle temporal cortex (Bonda et al. 1996) and the right portions of the parietal (Bonda et al. 1996; Grèzes et al. 2001) and frontal cortices (Saygin 2004). Bonda et al. (1996) also reported a bilateral involvement of the amygdala, which appears to be highly connected with the temporal cortex in monkeys (Aggleton et al. 1980; Amaral and Price, 1984). More recently, Sokolov et al. (2010) demonstrated the involvement of the left cerebellum in the perception of biological motion and delivered the first evidence for reciprocal communication between the left lateral cerebellum and the right posterior superior temporal sulcus (Sokolov et al. 2012).

In parallel to the studies using point-light displays, others have used simple geometric shapes to investigate the neural circuits underlying animacy. Unfortunately, in most of those studies, the animacy cues were mixed together (self-propulsion, spatiotemporal contingencies, directionality, etc.) which created complex motion designs that confound animacy detection with the detection of related social properties, such as intentions (for meta-analyses, see Molenberghs et al. 2012 and Van Overwalle and Baetens 2009). Moreover, the participants' attention was explicitly drawn to the mental states of the patterns, which might have affected the brain activation pattern. Blakemore et al. (2003) started to cope with those issues and tried to disentangle the neural correlates of animate perception, focusing only on spatiotemporal contingencies and self-propulsion. While the right lingual gyrus (bordering the medial fusiform gyrus) was specifically activated in response to a self-propelled agent, the left cerebellar cortex and the superior parietal lobe (bilaterally) were specifically activated in response to spatiotemporal contingencies. Differently from Blakemore et al. (2003), Stosic et al. (2014) used a single object jumping over a fence on its own to investigate the brain regions associated with self-propulsion and found an involvement of the inferior parietal lobe and the premotor cortex (also known as the mirror system, see Denny et al. 2012; Van Overwalle and Baetens 2009). Recently, Schultz and Bulthoof (2019) have also investigated the neural correlates associated with animacy perception. These authors placed a single dot in motion, manipulated its degree of perceived animacy (though self-propulsion) and found that the latest correlates with an intraparietal region: the right intraparietal sulcus.

Taken together, studies using biological motion patterns and simple shapes that drive animacy perception in humans seem to suggest a specialised neural network, where the right posterior superior temporal sulcus is a crucial node. One could then ask: are specific brain circuits responsive to animacy cues at birth? Some recent studies carried out in domestic chicks might answer this question. Mayer and colleagues have studied how the chick’s brain responds to conspecifics by measuring neural activation with the use of the immediate early gene c-Fos (Mayer et al. 2017a, b; Mayer et al. 2017a, b). They found higher activity in the septum and preoptic areas of chicks that encountered a live conspecific; both are key nodes of the so-called Social Behaviour Network (Newman 1999) of birds and mammals (O’Connell and Hofmann 2011). Later, Lorenzi et al. (2017) wondered whether the same regions would be involved if naïve chicks were exposed to the motion of a simple object driving animacy perception, such as a changing-speed disc (as depicted in Fig. 1b). Remarkably, the authors found higher brain activity in the septum and preoptic areas suggesting their involvement in animacy perception based on motion cues. Interestingly enough, those brain regions are also affected by substances controlling the social behaviour of naïve domestic chicks, such as mesotocin (the avian homologue of mammalian oxytocin, whose injection enhances the perceived salience of social stimuli soon after hatching, Loveland et al. 2019) or valproic acid (whose injection disrupts spontaneous social predispositions, Sgadò et al. 2018) a substance which is known to be a risk factor for autism spectrum disorder. These findings in domestic chicks suggest that the neural correlates of animacy perception are already functional before receiving specific experience.

## For a better understanding of human typical and atypical cognitive development

Although vertebrate species appear to be equipped with brain mechanisms sensitive to moving animacy cues, developmental processes and specific experience can influence and modulate them too (see Pavlova 2012 for a comprehensive review). For example, species and actions depicted by biological motion patterns (a walking person, dog, and a bird) are well-recognized in 3-year-old infants but steadily improve with time as 5-year-old infants appear to perform better (Pavlova et al. 2001). After this age, performance at detecting biological motion actions remains steady in adults and the elderly (Norman et al. 2004; Pavlova et al. 2001). Notably, the preference for looking at chasing events (using simple geometrical stimuli) also changes with age. 3–4-month-old infants preferentially look at the chasing events, while 5–6-month-old look more at the independent (random) moving of the elements (Rochat et al. 1997). These findings suggest an effect of experience on animacy perception that could be explained by a maturation of the neural networks at play. As a matter of fact, the perception of biological motion shows higher activation of the right posterior temporal sulcus (which is, as described in the previous section, heavily involved in the processing of visual information about animacy and intentions of others revealed by motion cues; Pelphrey and Carter 2008) and lower activation of the right fusiform gyrus in adults compared to 5–7-year-old (Lichtensteiger et al. 2008). Further studies carried out in children with social deficits, such as autism, provide additional insights into animacy perception and its mechanisms. Indeed, 1–12-year-old autistic infants are particularly impaired in processing biological motion and prefer to focus on non-social contingencies within the stimuli compared to the controls (Annaz et al. 2010; Klin et al. 2009; Klin and Jones 2008). However, adults with autism seem to be equally able as neurotypical controls to detect biological motion patterns (Murphy et al. 2009). Still, the neural networks function differently, as autistic individuals show a decreased activation in the right posterior temporal sulcus (Freitag et al., 2008) and the fusiform gyrus while watching biological motion patterns (Kaiser et al. 2010), which is probably one physiological cause of their social cognition impairments. This is a physiological impairment that could arise early in development if one links the research performed in humans and domestic chick. Indeed, it has recently been found that chick embryos injected with chemicals that are risk factors for autism spectrum disorder lost their preference for static and dynamic animacy cues (Lorenzi et al. 2019; Matsushima et al. 2022; Sgadò et al. 2018). In the same vein, human newborns and infants with low and high risks for autism perceive social stimuli differently (Di Giorgio et al. 2016, 2021a, b). Compared to low-risk controls, high-risk newborns were more likely to look at nonanimate stimuli. Interestingly, in 4 months, the pattern was reversed as high-risk infants looked more at the stimuli representing animacy than low-risk infants did (Di Giorgio et al. 2021a, b). This is particularly intriguing as it suggests that the deficit in social predispositions might not be caused by the absence of an adequate mechanism but by a development delay in its activation. It is important to note here that social predispositions are not available throughout the lifespan of an animal and have sensitive periods controlled by specific hormones, such as the thyroid hormone T3 (Lorenzi et al. 2021; Rosa-Salva et al. 2021). This is apparent in domestic chicks that prefer changing-speed stimuli just after hatching but not 3 days after (Lorenzi et al. 2021; Versace et al. 2019). However, when injected with the thyroid hormone T3, the preference for changing speed objects re-emerges in 3-day-old chicks (Lorenzi et al. 2021). This specific deficit to attend to animacy cues very early in the development might be a good indicator of social cognition disorders. Henceforth, the possibility of developing simple tests for early diagnosis of these neurodevelopmental disorders modelled on research on the humble domestic chick may prove feasible. An example can be found in research focusing on static (and more specifically, face-like patterns) rather than dynamic animacy cues. Indeed, using EEG and slow oscillatory visual stimulation, Buiatti and colleagues (2019) identified that the neural responses specific to face-like patterns of newborns overlap with those of adults. However, whether the neural correlates responding to animate motion (like self-propulsion or changing-states stimuli) is similar, or changes in human newborns and adults remain to be investigated. In line with those findings, both developmental and comparative research on animacy perception appears fundamental in nature but as we have seen, can also have profound and practical implications for human health.

## Conclusions

The development of animacy perception has been under scientific scrutiny for quite some time. Scientists have pondered whether animacy perception developed only as a result of one’s species-specific experience or if, instead, predisposed mechanisms have been shaped through evolution to canalise one’s attention toward non-species-specific cues that are typical of living animals (Lorenzi and Vallortigara 2021; Reid and Striano 2007; Vallortigara 2021). The empirical evidence presented in this review, taking advantage of domestic chicks and their precocial behaviours, favours the second hypothesis. Naive animals, namely, newly hatched chicks and human newborns, show biased attention toward specific motion cues common to most living organisms, and those naive biases are likely encoded by common neural mechanisms shaped by reciprocal interactions between social experience and genetic information (see Vallortigara and Rosa-Salva, 2017 for a comprehensive review). Researchers, now, need to elucidate how and in what form those social predispositions are encoded in the brain. Is animacy perception encoded at the level of single neurons responding to specific configurations (such as self-propulsion, biological motion, directionality, etc., see chapter 19 of Vallortigara 2021) or in more complex pre-wired circuits passed by a set of general rules integrated into a genomic bottleneck (see Koulakov et al. 2022 and Zador 2019)? What are the genetic mechanisms underlying animacy perception and to what extent can they be modulated by experience? These are some of the issues on the agenda for future research.
